# AOViT: an adaptive optimized vision transformer model for the classification of heritage paintings

**DOI:** 10.3389/frai.2026.1950812

**Published:** 2026-10-26

**Authors:** Bassam Noori Shaker, Lafta Raheem Ali, Ammar T. Namel, Sam Ansari, Dina Shehada, Shadi Atalla, Abir Jaafar Hussain

**Affiliations:** 1 Computer Science Department, College of Computer Science and Information Technology, University of Al-Qadisiyah, Al Diwaniyah, Iraq; 2 General Directorate of Education of Salahuddin, Tikrit, Salahuddin, Iraq; 3 General Directorate of Education in Wasit, Wasit, Iraq; 4 Research Institute of Sciences and Engineering, University of Sharjah, Sharjah, United Arab Emirates; 5 Department of Electrical Engineering, College of Engineering, University of Sharjah, Sharjah, United Arab Emirates; 6 College of Engineering & IT, University of Dubai, Dubai, United Arab Emirates; 7 School of Computer Science and Mathematics, Liverpool John Moores University, Liverpool, United Kingdom

**Keywords:** AdamW optimizer, artistic style classification, learning rate scheduler, self-attention, vision transformer (ViT)

## Abstract

The automated classification of artistic styles in paintings is a fundamental task in digital art history and computational aesthetics. For years, the dominant paradigm for this image-recognition problem has relied on convolutional neural networks (CNNs), including architectures such as VGG16, VGG19, Inception, EfficientNet, and ResNet, whose inductive biases for local feature extraction have yielded strong performance. However, this reliance on local processing may limit a model's ability to capture global stylistic cues, such as composition, brushwork patterns, and holistic texture, that are often critical for distinguishing artistic movements. To address this limitation, this study proposes AOViT, a Vision Transformer (ViT)-based model tailored to artistic style classification. By exploiting the self-attention mechanism of the ViT architecture, the proposed model captures long-range dependencies and global contextual information. Experimental evaluation on the Persian Miniatures dataset yielded a mean accuracy of 93.85% under five-fold cross-validation. Furthermore, the results indicate that AOViT provides a competitive alternative to CNN-based models for artistic style classification, particularly when combined with appropriate training strategies under limited-data conditions.

## 1 Introduction

The intersection of computer vision and art history has created a vibrant field of research, where the automated analysis of visual art presents unique challenges and opportunities. A central task within this domain is the classification of paintings by artistic style, a label that encapsulates the shared techniques, philosophies, and visual idioms of a movement, such as Impressionism, Cubism, or Baroque. This task is crucial for applications ranging from museum curation and art education to the large-scale analysis of cultural heritage collections (Foka, 2021).

Traditionally, this challenge has been addressed using Convolutional Neural Networks (CNNs), which can extract local features like edges, textures, and shapes (Ali et al., 2025). While successful, CNNs are fundamentally designed to process information in a local, progressive manner. This architectural bias may act as a bottleneck for capturing the non-local and often abstract relationships that define an artistic style. The defining elements of a style—such as the dynamic composition of a Baroque piece, the expansive blended skies of Impressionism, or the fragmented geometry of Cubism—require a model capable of integrating information across the entire image from the outset to capture the work's overall visual structure (Castellano and Vessio, 2021). Despite their success, artistic style classification remains challenging due to the need to capture both local details and global stylistic patterns, particularly when working with limited and imbalanced datasets.

Recently, the Vision Transformer (ViT) architecture has emerged as a powerful alternative to CNNs, challenging long-held assumptions about how machines should process images. By treating an image as a sequence of patches and employing a self-attention mechanism, ViT models can capture global dependencies between all patches simultaneously, regardless of their spatial proximity (Kameswari et al., 2023). This capability for global contextual integration makes ViT particularly suitable for the macro-scale analysis required in art style recognition, where overall composition and long-range interactions of visual elements are important (Wang et al., 2025).

However, the effectiveness of Vision Transformers is highly dependent on large-scale training datasets. This requirement presents a significant limitation in the domain of artistic style classification, particularly for historical and cultural heritage datasets, which are typically small due to the fixed and irreplaceable nature of artworks (Han et al., 2023). A primary solution to this challenge is data augmentation (Alomar et al., 2023). This process artificially expands the dataset by generating modified copies of existing images through transformations such as flipping, rotation, resizing, and shearing. Consequently, the increased volume of training data facilitates a more effective learning process and enhances the model's overall performance (Lewy and Mańdziuk, 2023).

Furthermore, various optimization algorithms, such as Adam, stochastic gradient descent (SGD), and AdaDelta, can be used to update model weights and improve generalization. Among their key hyperparameters, the learning rate plays a central role in controlling the optimization process and influencing convergence behavior (Wani et al., 2020). In addition, AdamW was developed as a modification of Adam that decouples weight decay from the gradient-based parameter update, thereby providing more principled regularization (Orabona, 2022). Researchers have also moved beyond AdamW to develop learning rate schedulers, which dynamically adjust the learning rate during training. This adaptation leads to improved convergence, generalization, and stability (Park et al., 2020).

To improve the interpretability of deep-learning models in image-based tasks, Grad-CAM can be applied. This widely used explainable artificial intelligence (XAI) technique generates a heatmap that highlights image regions that contribute strongly to a model's prediction. The heatmap can then be superimposed on the original image to visualize the regions most influential to the model's decision (Selvaraju et al., 2016). This process provides insight into the model's decision-making behavior by identifying image regions associated with influential features. Consequently, Grad-CAM can improve model transparency and support qualitative inspection and debugging of classification or detection systems (Saporta et al., 2022).

In this study, we develop AOViT, a task-adapted ViT framework for artistic style classification. The proposed approach uses self-attention to model global relationships among image patches and combines transfer learning, data augmentation, AdamW optimization, and learning-rate scheduling within a structured training pipeline. The model is evaluated on Persian miniature paintings using five-fold cross-validation and is compared with several CNN-based and hybrid architectures. In addition, Grad-CAM is used to provide qualitative insight into the image regions that influence the model's predictions. The main contributions of this study are:

- A task-specific adaptation of Vision Transformers for artistic style classification under limited data conditions.
- A structured training pipeline integrating transfer learning, data augmentation, AdamW optimization, and learning rate scheduling to improve generalization.

The rest of this paper is organized as follows. In Section 2, a literature review of the related research on artistic style classification is presented. Section 3 briefly outlines the theoretical approaches of the techniques used in the current study. Section 4 describes the proposed methodology, including the dataset, AOViT architecture, and experimental setup. Section 5 reports the experimental and comparative results, including evaluations against several CNN and hybrid models. Finally, Section 6 summarizes the main findings, discusses the study limitations, and outlines directions for future research.

## 2 Related work

Painting-style classification relies on visual features that characterize the artwork. Feature-extraction methods have evolved from handcrafted descriptors to modern machine-learning and deep-learning approaches. Among the most widely used deep-learning methods are CNNs, which are effective at learning local features associated with stylistic cues such as brushstrokes, textures, and color patterns, thereby supporting painting-style classification and artist identification.

Gatys et al. (2016) introduced a CNN-based framework that used Gram-matrix representations derived from a pre-trained VGG-19 network to model artistic style, establishing an influential foundation for subsequent approaches. Despite these advances, CNN-based methods can remain limited by their emphasis on local receptive fields when global compositional relationships are important for distinguishing artistic styles.

Other researchers have sought to enhance CNN performance by incorporating specialized data modalities. Kim et al. (2019) proposed a new data utilization approach in machine learning with Reflectance Transformation Imaging (RTI), which maximizes the visualization of a three-dimensional shape of brushstrokes. Their experiments showed an RTI-trained ResNet-50 model achieving 87.43% accuracy. However, this approach requires specialized RTI capture equipment that limits its scalability to existing digital art collections.

Moving beyond pure visual analysis, Garcia et al. (2020) proposed a model to capture contextual artistic information from fine-art paintings with a specific ContextNet network. As context can be obtained from multiple sources, they explore two modalities of ContextNets: one based on multitask learning and another one based on knowledge graphs. Once the contextual information is obtained, they use it to enhance visual representations computed with a neural network. Based on evaluations of classification and retrieval tasks, together with graph visualizations, the authors reported that their method successfully captured distinct stylistic aspects. However, its implementation requires extensive structured metadata to build the knowledge graph, which is not always available.

Researchers have also extended CNN architectures for artistic stylizations. Chen et al. (2021a) proposed a novel internal-external learning framework for style transfer that achieved a 26.7% user preference score and a 62.4% deception score. However, this method uses a complex Generative Adversarial Network (GAN) framework designed for image generation rather than direct style classification.

The flexibility of CNN frameworks is further demonstrated in domain-specific applications. Chen (2022) developed a method for classifying Chinese paintings by fine-tuning VGG-F CNN and introducing an embedded classification algorithm based on mutual information, achieving 94.88% mean precision. However, this approach struggles with artists whose style is highly eclectic or derivative and is limited to small, domain-specific datasets.

Building on this, Ugail et al. (2023) applied deep transfer learning using ResNet50 for feature extraction combined with a Support Vector Machine (SVM) classifier for binary authentication of Raphael's paintings. These features were integrated with traditional edge-detection descriptors (Canny, Sobel, Laplacian, and Scharr) to capture brushstroke signatures. Although the authors achieved 98% accuracy, their approach is limited to artist-specific authentication and requires manual feature engineering for edge detection.

Parallel to these advancements, Imran et al. (2023) proposed a classification system with two phases. In the first phase, a CNN was used for patch analysis; in the second phase, a shallow neural network operated on the resulting representations, achieving 96.5% accuracy on the Australian Native Art dataset. Nevertheless, it should be noted that this two-stage system is computationally inefficient for processing single images.

Menis-Mastromichalakis et al. (2024) developed sophisticated ensemble approaches by conducting a comparative study of eight CNN architectures on the WikiArt and Pandora 18K datasets. They proposed a stacking ensemble that combined predictions from these architectures using a meta-classifier and achieved 68.55% accuracy on the challenging WikiArt dataset. However, this ensemble approach is computationally expensive and lacks a unified architecture.

Subsequent work incorporated attention mechanisms into CNN backbones. Li et al. (2024) added a Pyramid Spatial Attention (PSA) module and reported that the model could capture both local detail and global structure by dynamically reweighting feature maps across spatial locations and scales. On the WikiArt dataset, the model achieved approximately 91.52% accuracy. However, the attention mechanism remained an auxiliary module attached to a CNN backbone primarily designed for local feature extraction.

For Persian miniature classification, Shahi et al. (2025) proposed a CNN-based method using a curated dataset. A patch-based CNN independently classified image patches and subsequently fused their predictions, yielding an average accuracy above 91%. However, because the method processes patches independently before late-stage fusion, it may capture global compositional context less directly than architectures that model long-range interactions throughout the image.

Su et al. (2025) introduced a prototypical network-based solution for material classification in images of traditional Chinese paintings, which is based on ResNet18 backbone as well as cropping-based augmentation and multitask optimization. According to the authors, their work manages to effectively preserve fine-grained local features like brushstroke textures, and thereby enhances generalization through auxiliary tasks such as dynasty classification, especially in a few-shot setting. The model was able to reach an accuracy of around 80% after training with just 30 samples per class, demonstrating its effectiveness in low-data settings. The proposed method still uses a CNN backbone, which is mainly developed for local feature extraction, and may not fully model long-range dependencies and global contextual relationships.

Finally, to address culturally specific art forms, Xu and Lu (2026) presented a multi-stage framework for artistic style classification by integrating the local and global feature learning. Their method first divides each painting into meaningful visual areas by means of K-means clustering, which allows them to extract features in localized areas. Each region is processed by multiple CNNs to learn fine-grained representations, while an additional deeper CNN is applied to the entire image to capture global contextual information. They subsequently fuse the local and global features using a self-attention mechanism that dynamically emphasizes the most informative regions. An autoencoder is then employed to reduce dimensionality and suppress noise before classification using a Softmax layer. The method achieved 89.07% accuracy on the MET dataset, but its multi-network structure increases computational complexity, and its reliance on a fixed clustering strategy may limit adaptability to highly diverse or complex artistic images. The various methodologies, datasets, and limitations of reviewed state-of-the-art works are shown in Table 1.

**Table 1:** Summary of related work.

Reference	Methodology	Dataset	Performance Metric	Limitations
Gatys et al. (2016)	A style transfer framework based on a pre-trained VGG CNN.	Arbitrary photographs + famous artworks (e.g., Van Gogh, Picasso, Kandinsky).	Qualitative visual assessment	Relies on CNNs with local receptive fields, limiting the ability to capture global compositional structure in artistic styles.
Kim et al. (2019)	CNN trained on RTI data.	Proprietary oil paintings	Accuracy: 87.43%	Relies on specialized hardware (RTI) not available for existing digital collections, limiting scalability.
Garcia et al. (2020)	ContextNet: uses multi-task learning (MTL) and a knowledge fraph (KG).	SemArt	Accuracy: 81.5%	Requires extensive structured metadata to build the knowledge graph, which is not always available. Dependency on explicit metadata.
Chen et al. (2021a)	Internal-external learning (GAN) + contrastive losses.	MS-COCO, WikiArt	Qualitative visual assessment	The method is a complex GAN-based framework for image generation/transfer rather than direct style classification. It requires adversarial training.
Chen (2022)	Fine-tuned VGG-F with feature weighting via mutual information.	Chinese Paintings	Precision: 94.88%	Performance is limited to a small, domain-specific dataset and struggles with eclectic styles.
Ugail et al. (2023)	Transfer learning (ResNet50) + SVM; edge detection.	Raphael paintings	Accuracy: 98%	The model is specific to a single artist (Raphael) and requires handcrafted edge analysis.
Imran et al. (2023)	Two-phase: Deep CNN on patches + Shallow NN on probability vectors.	WikiArt, Australian Art	Accuracy: 96.5%	The two-stage pipeline is complex and computationally inefficient for processing a single image.
Menis-Mastromichalakis et al. (2024)	Comparative study of eight CNN architectures; stacking ensemble.	WikiArt, Pandora 18K	Accuracy: 68.55%	Complex multi-model ensembles are computationally expensive and lack a unified architecture.
Li et al. (2024)	CNN with Pyramid Spatial Attention (PSA) module.	WikiArt	Accuracy: 91.52%	The attention mechanism is a secondary module added to a CNN designed primarily for local feature extraction.
Shahi et al. (2025)	Patch-based classification using DenseNet; prediction fusion.	Persian Miniatures	Accuracy: 91%	The patch-based approach processes image segments independently before late-stage fusion, potentially losing the global compositional context.
Su et al. (2025)	CNN + Few-shot learning	Self-constructed Chinese painting dataset	~80% (30 samples/class)	Relies on a CNN backbone, limiting global context modeling.
Xu and Lu (2026)	Hybrid framework integrating K-means clustering, CNNs, self-attention, and autoencoder.	MET	Accuracy: 89.07%	High computational complexity due to multiple CNN architectures and feature fusion stages. Dependence on a predefined K-means clustering strategy may limit adaptability to highly diverse or complex artistic images.

The reviewed studies reveal several recurring limitations, including computationally complex ensemble or multi-stage architectures and a strong dependence on CNN backbones that primarily emphasize local feature extraction. This work addresses these limitations by developing AOViT, a ViT-based framework for recognizing patterns in heritage paintings. Through self-attention, ViTs can model long-range dependencies across the image, enabling relationships among composition, texture, and color to be represented more directly when characterizing artistic styles or movements.

## 3 Theoretical background

Section 3 outlines the theoretical foundations of the proposed AOViT model for artistic style recognition. Three subsections are presented. Section 3.1 introduces artistic style recognition, Section 3.2 discusses visual painting-style characteristics such as brushstrokes, color, texture, and composition, and Section 3.4 explains the use of ViTs and self-attention for modeling global image dependencies. Together, these concepts provide the theoretical basis for painting analysis and classification.

### 3.1 Artistic style recognition

Artistic style recognition is a fundamental task in the intersection of computer vision and digital art analysis, aiming to automatically identify the stylistic attributes of paintings and artworks (Chen et al., 2021b). The concept of style in art extends beyond subject matter, encompassing visual elements such as brushstroke techniques and color schemes, which can help define the characteristics of a particular artist, school, or historical movement.

Early approaches to style recognition relied on handcrafted features, such as edge descriptors, color histograms, and texture filters, to capture the visual patterns present in artworks. While these methods provided useful insights, they were often limited in their ability to model the complex, abstract, and hierarchical nature of artistic styles. With the advent of deep learning, particularly CNNs, significant progress has been achieved by automatically extracting hierarchical features from paintings. CNN-based methods demonstrated strong performance in distinguishing between art movements and artists by learning discriminative representations directly from image data (Castellano and Vessio, 2021).

Despite these advancements, challenges remain in artistic style recognition. Paintings from the same style can exhibit high intra-class variability, while different styles may share overlapping features, making classification a non-trivial problem. These challenges motivate the exploration of more advanced architectures, such as ViTs, which can capture long-range dependencies and focus on subtle stylistic cues that are often overlooked by traditional convolutional models.

### 3.2 Painting style characteristics

Painting styles are defined by distinctive visual features that reflect the techniques, preferences, and cultural context of artists or artistic movements. Unlike general image recognition, which often relies on object shapes or semantic content, style recognition focuses on abstract and perceptual attributes embedded within the artwork. These include brushstroke patterns, texture, composition, and color usage, which together form the unique identity of a painting style (Srinivasan et al., 2024).

Brushstrokes serve as one of the most significant indicators of style, as their direction, density, and smoothness can highlight differences between artistic schools or even individual artists (Bigerelle et al., 2023). Similarly, texture contributes to style characterization, with variations in roughness, layering, and granularity offering clues about the materials and techniques employed. Color palettes also play a crucial role, as different movements often emphasize particular hues and contrasts to convey mood or symbolism. In addition, composition—the spatial arrangement of elements within the canvas—can distinguish styles, as seen in the structured balance of Renaissance art compared to the dynamic perspectives of Impressionism.

However, these stylistic attributes are not always straightforward to capture computationally. Paintings within the same style may display considerable variation, while stylistically distinct works can share overlapping features. This complexity highlights the importance of advanced feature extraction methods capable of capturing subtle stylistic nuances, such as those provided by ViT architectures.

### 3.3 Vision transformers

Recently, the landscape of computer vision has evolved significantly with the introduction of ViTs, which challenge the dominance of CNNs (Han et al., 2023; Huo and Cai, 2023). While CNNs have served as the backbone of many state-of-the-art image-classification models, ViTs have gained attention for their unique approach to processing visual data.

A ViT decomposes an input image into a series of non-overlapping, equal-sized groups of pixels called patches. These two-dimensional patches are flattened into one-dimensional vectors by concatenating their pixel values. The resulting vectors are then projected into an embedding space using a linear transformation, and positional information is added to preserve the spatial ordering of patches. Since the ViT has no inherent knowledge of the order, relative position, or dimensionality of the patches, these embeddings are crucial for encoding spatial relationships learned during the training process. The resulting sequence of encoded patch vectors is then fed into a standard transformer encoder (Kameswari et al., 2023).

A self-attention mechanism is employed to perform tasks similar to those of recurrent neural networks (RNNs). However, self-attention eliminates the computational repetition associated with processing previous hidden states. This mechanism also enhances computational efficiency by allowing parallel processing of all elements in the sequence. While CNNs are limited to a fixed local receptive field defined by their kernel size, self-attention excels by dynamically capturing features across a much wider range and generalizing these features (Wang et al., 2025).

In this study, the ViT architecture is employed as the backbone of the proposed recognition model because it is well suited to this task. ViT allows the model to concentrate on the most informative regions of a painting, such as distinctive brushstrokes, color schemes, and textural patterns that characterize a specific artist or artistic movement, while giving less importance to the depicted subject itself.

## 4 Methodology

Section 4 describes the implementation details of the proposed AOViT framework. The model follows a structured pipeline comprising data preprocessing, data augmentation, feature extraction and representation learning, model optimization, classification, and explainability analysis. Initially, all images are resized and normalized to ensure consistency in the input data. Data augmentation is then applied to the training data to increase sample diversity. The images are subsequently partitioned into fixed-size patches and processed by the Vision Transformer (ViT), where self-attention models relationships among patches and captures global contextual information relevant to stylistic classification. During training, the AdamW optimizer and a cosine annealing learning-rate scheduler are employed. After training, the model performs artistic-style classification, while Grad-CAM is used to provide visual interpretations of the model's decision-making process. Section 4.1 introduces the dataset and its key characteristics, Section 4.2 details the preprocessing procedures, and Section 4.3 presents the architecture and training workflow of the proposed AOViT model, as illustrated in Figure 1.

**Figure 1:**
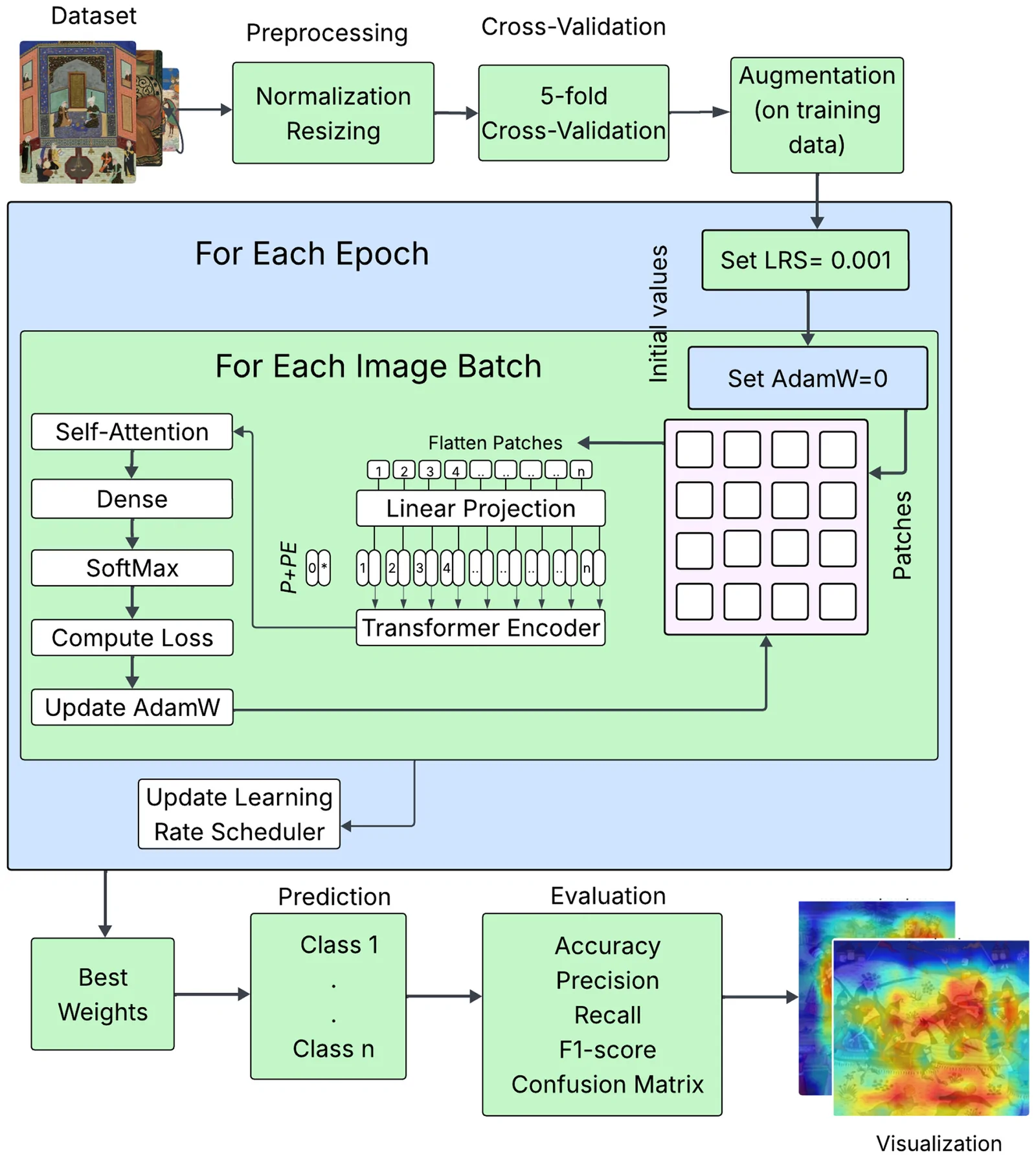
Overall workflow of the proposed AOViT framework, highlighting transfer learning, input-adaptive self-attention, and optimization through AdamW and cosine learning-rate scheduling.

In this context, the term “adaptive” does not refer to a novel attention module; rather, it signifies two characteristics: first, the self-attention mechanism calculates the weights of relationships between patches based on the specific content of the input image; and second, all model parameters are adapted–via fine-tuning–to the domain of Persian miniature paintings. The term “optimized” refers to the set of training strategies employed, including AdamW and cosine learning-rate scheduling. Therefore, AOViT should be interpreted as a task-adapted and optimization-oriented ViT framework, rather than the introduction of an entirely new Transformer block.

### 4.1 Dataset description

This study employs the Persian Miniature dataset (Shahi, 2025), a curated collection of 890 images of Persian miniature paintings categorized into five historical and stylistic schools. The distribution of images for each painting school is shown in Table 2. This dataset was recently used for CNN-based stylistic classification by Shahi et al. (2025). It was sourced from the Islamic Painted Page archive and refined through manual preprocessing, including cleaning, cropping, and removal of marginalia, to isolate the core artwork.

**Table 2:** Dataset class distribution.

School of art	Number of images	Percentage of dataset
Herat	202	22.7%
Qajar	150	16.9%
Shiraz-e Avval	193	21.7%
Tabriz-e Avval	190	21.3%
Tabriz-e Dovvom	155	17.4%
Total	890	100.0%

Compared to larger but unannotated museum archives, this dataset was selected due to its diverse class labels, high-quality curation, and prior validation in computational research, making it a reliable foundation for the classification and feature analysis objectives of this study. Figure 2 presents one representative sample from each dataset class.

**Figure 2:**
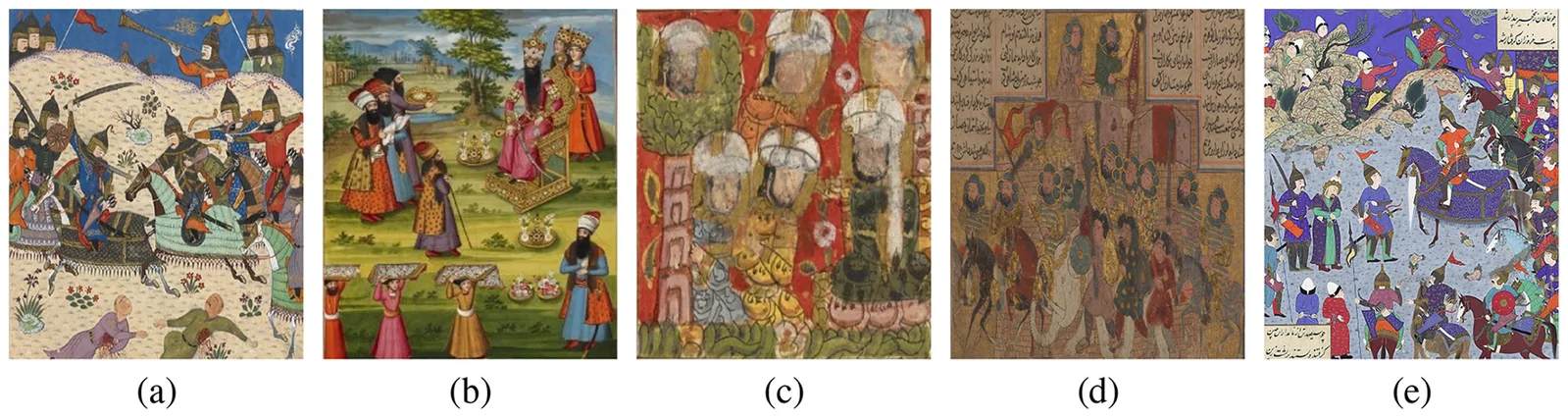
Samples from the dataset: **(a)** Herat, **(b)** Qajar, **(c)** Shiraz-e Avval, **(d)** Tabriz-e Avval, and **(e)** Tabriz-e Dovvom.

Despite its high-quality curation, the dataset is relatively small. This limited size introduces potential risks of overfitting and reduced generalization, particularly for transformer-based models that are commonly trained on large-scale datasets. Therefore, robust evaluation and training strategies, including five-fold cross-validation and training-set augmentation, were used to improve the reliability of the evaluation and increase the diversity of the training data.

### 4.2 Preprocessing

Several preprocessing operations were applied, including resizing, normalization, and data augmentation. Initially, all images were resized to 224 × 224 pixels to match the input requirements of the ViT model. This resizing established a standardized input format for model training and comparison.

The next preprocessing step addressed differences in feature scale using *Z*-score normalization. This transformation standardizes the input values to have a mean of zero and a standard deviation of one, which can improve numerical stability during optimization. Equation 1 defines the *Z*-score normalization applied to the input images.


$$\begin{array}{l} z_{ij}=\frac{a_{ij}-\mu }{\sigma } \end{array}$$ (1)

where *z*_*ij*_ denotes the normalized value at pixel position (*i, j*), *a*_*ij*_ is the corresponding original value, and μ and σ denote the mean and standard deviation, respectively.

To ensure robust evaluation and reduce dependence on a single data split, five-fold cross-validation was employed. Data augmentation was applied only to the training folds to increase training-sample diversity and expose the model to controlled image transformations. Table 3 lists the augmentation techniques used and their corresponding values.

**Table 3:** Data augmentation configuration.

Technique	Value
Left and right flip	0.5
Rotation	(−15°, +15°)
Shear	10°
Brightness	0.1
Contrast	0.1
Saturation	0.1

### 4.3 The proposed model training process

At the beginning of the training process, the model hyperparameters were initialized. The values used in the experiments are listed in Table 4.

**Table 4:** ViT model hyperparameters.

Hyperparameter	Value
Epochs	20
Initial learning rate	0.001
Optimizer	AdamW
Scheduler	Cosine annealing
Scheduler update	Once per epoch
Batch size	8
Weight decay	1 × 10^−4^
Patch size	16
Attention heads	3

#### 4.3.1 Architectural details and reproducibility

The proposed AOViT model was developed based on a lightweight version of the ViT, specifically the ViT-Tiny/16 configuration. Input images were first resized to 224 × 224 pixels and then divided into non-overlapping 16 × 16 patches. Consequently, each image was converted into 196 patches; following a linear projection, each patch was transformed into a latent vector with a dimensionality of 192. A learnable classification token was prepended to the sequence of patches, and positional embeddings were added to the input tokens.

The Transformer core consists of 12 sequential encoder blocks. Each block comprises a multi-head self-attention module with three attention heads, normalization layers, residual connections, and a GELU-based feed-forward network. Compared to ViT-Base, this lightweight design substantially reduces the number of parameters and computational cost, making it more suitable for the relatively small dataset used in this study.

To mitigate the risk of overfitting and leverage previously learned visual representations, the backbone was instantiated using the *timm* model identifier vit_tiny_patch16_224 with pretrained=True. The original classification head was replaced with an output layer tailored to the dataset's five classes, and all trainable network parameters were subsequently updated during the fine-tuning phase. In other words, the backbone was not frozen, and fine-tuning was conducted end-to-end. The original experimental environment did not explicitly record the exact *timm* package version or the internal checkpoint metadata; therefore, the manuscript reports the model identifier and initialization flag that were used, rather than assigning an unverified pretraining corpus or checkpoint name.

Model training was performed using the categorical cross-entropy loss function. Parameter optimization utilized AdamW with weight decay, and the learning rate was adjusted during training via cosine annealing. All primary experiments were conducted within a five-fold cross-validation framework. In each iteration, four-fold were used for training and the remaining fold for evaluation; this process was repeated until each fold had served as the evaluation set exactly once. The final results were reported as the mean and standard deviation of the performance across the five-fold. To avoid using an evaluation fold for epoch selection, training was run for the predefined 20 epochs and the checkpoint from the final epoch was retained for fold-wise evaluation; no “best epoch” was selected using the evaluation fold. To enhance methodological transparency and reproducibility, the full architectural configuration and key implementation details of the AOViT model are presented in Table 5.

**Table 5:** Architectural configuration and implementation details of the proposed AOViT model.

Architectural component	Configuration
Backbone	ViT-Tiny/16
Input resolution	224 × 224
Patch size	16 × 16
Number of patches	196
Embedding dimension	192
Transformer encoder blocks	12
Attention heads	3
MLP ratio	4
Activation	GELU
Classification token	Learnable CLS token
Positional embedding	Learnable positional embedding
Pretraining	*timm* default pretrained weights
Model identifier	vit_tiny_patch16_224
Initialization flag	pretrained=True
Fine-tuning	End-to-end
Number of classes	5
Loss function	Cross-entropy
Optimizer	AdamW
Scheduler	Cosine annealing
Cross-validation	five-fold
Trainable parameters	5.53 M

In this study, the ViT-Tiny/16 configuration was employed as the primary backbone. This architecture divides 224 × 224 images into 16 × 16 patches and utilizes an embedding dimension of 192, 12 encoder blocks, and three attention heads. The Tiny version was selected over ViT-Base to reduce the number of parameters, computational cost, and the risk of overfitting given the small size of the dataset. With this configuration and a five-class classification head, the model has approximately 5.53 million trainable parameters—substantially fewer than larger ViT variants. This lightweight configuration was selected to reduce computational complexity and mitigate the risk of overfitting on the limited dataset used in this study.

A cosine annealing learning-rate scheduler was used to adjust the learning rate during training according to Equation 2. The scheduler was advanced once after each complete training epoch, rather than after each mini-batch; therefore, the scheduler position *T*_cur_ is interpreted on an epoch basis in this implementation.


$$\begin{array}{c} \eta_{t}=\eta_{\min}^{i}+\frac{1}{2}\left(\eta_{\max}^{i}-\eta_{\min}^{i}\right)\left(1+\cos\left(\frac{T_{\text{cur}}}{T_{i}}\pi \right)\right) \end{array}$$ (2)

where $\eta_{\min}^{i}$ and $\eta_{\max}^{i}$ are the minimum and maximum learning-rate bounds, respectively, *T*_cur_ denotes the current scheduler position, and *T*_*i*_ denotes the corresponding cosine-cycle length.

At the beginning of a cosine cycle, when *T*_cur_ = 0, the learning rate η_*t*_ is equal to $\eta_{\max}^{i}$. As *T*_cur_ approaches *T*_*i*_, the cosine term decreases to −1, and the learning rate approaches $\eta_{\min}^{i}$. In the present training protocol, this update is performed at the end of each epoch, which is consistent with the epoch-level update shown in Algorithm 1.

Algorithm 1 AOViT architecture, data preparation, and fold initialization.

 **Require:**  Dataset *D* containing five Persian miniature
  painting classes
**Ensure:**  Initialized training configuration for each
  cross-validation fold
1:  **Initialize AOViT architecture:**
      Input resolution *H*×*W* = 224 × 224
      Patch size *P* = 16
      Number of patches *N* = 196
      Embedding dimension *E* = 192
      Transformer encoder layers *L* = 12
      Attention heads *AH* = 3
      Number of output classes *C* = 5
2:  **Initialize training configuration:**
      Initial learning rate *LRS* = 0.001
      Batch size *B* = 8
      Weight decay = 1 × 10^−4^
      Number of epochs = 20
      Random seed = 42
3:  Resize all images to 224 × 224
4:  Normalize input images using Equation 1
5:  Partition dataset *D* into five-fold:
      {*D*_1_, *D*_2_, *D*_3_, *D*_4_, *D*_5_}
6:  **for** *k* = 1 to 5 **do**
7:     Set *D*_*k*_ as the evaluation fold
8:     Use the remaining four-fold as the training set
9:     Apply data augmentation only to training
   images:
      Horizontal flip probability = 0.5
      Rotation = (−15°, +15°)
      Shear = 10°
      Brightness adjustment = 0.1
      Contrast adjustment = 0.1
      Saturation adjustment = 0.1
10:   Instantiate vit_tiny_patch16_224 from *timm*
11:   Set pretrained=True to load the default
  pretrained weights
12:   Replace the original classification head with
  a five-class output layer
13:     Enable end-to-end fine-tuning of all trainable
  parameters
14:    Initialize AdamW optimizer
15:    Initialize cosine annealing learning-rate
  scheduler for epoch-level updates
16:    Continue training according to Algorithm 1
17:  **end for**

At this stage, the ViT module divides each input image into non-overlapping 16 × 16 patches, resulting in 196 patches arranged in a 14 × 14 grid because 224/16 = 14. Equation 3 defines the resulting number of patches.


$$\begin{array}{c} N=\frac{H\times W}{P^{2}}, \end{array}$$ (3)

where *N* is the number of patches, *H* and *W* denote the image height and width, respectively, and *P* is the patch dimension.

The AdamW optimizer updates the model parameters after each mini-batch of eight images, as expressed in Equation 4.


$$\begin{array}{c} w_{t}=\left(1-\eta_{t}\lambda \right)w_{t-1}-\eta_{t}\frac{{\hat{m}}_{t}}{\sqrt{{\hat{v}}_{t}}+\epsilon }, \end{array}$$ (4)

where *w*_*t*_ represents the network's weights, λ is the weight decay factor, ${\hat{m}}_{t}$ and ${\hat{v}}_{t}$ are the bias-corrected estimates of the first and second moments (gradient and squared gradient), respectively, and ϵ is a small constant added for numerical stability.

#### 4.3.2 Transfer learning and fine-tuning strategy

Given the limited size of the dataset, training a Vision Transformer from scratch (random initialization) could increase the risk of overfitting and optimization instability. Therefore, the proposed framework employed transfer learning. The ViT-Tiny/16 backbone was instantiated through *timm* using the model identifier vit_tiny_patch16_224 with pretrained=True, after which the original classification head was replaced with an output layer tailored to the five target classes. Because the original environment did not pin the *timm* version or log the internal pretrained-checkpoint metadata, no more specific pretraining dataset or checkpoint designation is asserted here.

Unlike a fixed feature extraction approach, the backbone was not frozen in this study; instead, all trainable model parameters—including the Transformer encoder layers and the new classification head—were updated end-to-end during the fine-tuning phase. This strategy leveraged the general visual representations learned during pre-training while simultaneously allowing the model parameters to adapt to the specific characteristics of Persian miniature paintings, such as composition, texture, color patterns, and global relationships between visual elements.

Transfer learning was particularly appropriate for the limited dataset because initialization from pre-trained weights provides a structured starting point and reduces the need to learn visual representations entirely from scratch. Model performance was nevertheless evaluated using five-fold cross-validation to reduce dependence on any single data split.

#### 4.3.3 Linear projection

Transformers were originally designed for natural language processing and relied on word embeddings. To adapt this architecture for images, a new mechanism was needed to convert pixel data into a similar, embedding-like format. The ViT achieves this by first splitting an image into fixed-size patches. These patches are flattened and projected by a linear layer into fixed-dimensional embedding vectors. This linear projection acts as a critical bridge, translating visual data into a form processable by the Transformer architecture. An image **x**∈ℝ^*H*×*W*×*C*^, where *C* denotes the number of channels (e.g., 3 for an RGB image), is transformed into a sequence of patches ${\text{x}}_{p}\in {\mathbb{R}}^{N\times \left(P^{2}\times C\right)}$, which can then be projected into the Transformer embedding space.

As each image is divided into a sequence of 196 patches, spatial order must be encoded explicitly because the Transformer does not otherwise preserve the two-dimensional arrangement of the image patches. Consistent with the ViT-Tiny/16 implementation used in this study, a learnable positional-embedding matrix is added to the token sequence. These positional parameters are optimized jointly with the remaining model parameters during end-to-end fine-tuning, allowing the network to learn position-dependent representations directly from the data.

For each patch, an embedding vector Embd_*i*_ is generated by applying a learnable projection matrix *W*_Embd_ to the patch representation *x*_*i*_, as shown in Equation 5.


$$\begin{array}{c} \text{Emb}{\text{d}}_{i}=x_{i}\cdot W_{\text{Embd}}. \end{array}$$ (5)

Second, a special learnable vector, referred to as the class (CLS) token, is prepended to the patch sequence.

Thus, after these processing steps, the ViT constructs the final input sequence by prepending a learnable class token to the sequence of patch embeddings and adding a learnable positional vector to each token, including the CLS token. This complete sequence is subsequently fed into the Transformer encoder. Equation 6 shows the resulting input sequence.


$$\begin{array}{c} z_{0}=\left[x_{\text{class}}+P_{\text{class}},\text{Emb}{\text{d}}_{1}+P_{1},\text{Emb}{\text{d}}_{2}+P_{2},\ldots ,\text{Emb}{\text{d}}_{N}+P_{N}\right]. \end{array}$$ (6)

As this sequence is processed through the Transformer's self-attention mechanism, the CLS token aggregates global information from all patches. The final CLS-token representation summarizes information aggregated from the patch sequence and is used by the classification head to generate the final prediction.

#### 4.3.4 Transformer encoder

The Transformer encoder processes the patch sequence through 12 encoder blocks. Each block contains a multi-head self-attention (MHSA) module with three attention heads and a position-wise feed-forward network (FFN). The FFN uses the Gaussian Error Linear Unit (GELU) activation function, applied independently to each token between fully connected layers. Residual connections and normalization layers are used to support stable optimization and gradient flow. The GELU activation for an input *x* is approximated by Equation 7.


$$\begin{array}{c} \text{GELU}\left(x\right)\approx 0.5x\left(1+\tanh\left[\sqrt{\frac{2}{\text{π}}}\left(x+0.044715x^{3}\right)\right]\right). \end{array}$$ (7)

The encoder refines the embeddings by repeatedly integrating information across the entire image through each successive layer. The final output is a sequence of embeddings that captures both fine-grained local details and a rich global context.

#### 4.3.5 ViT self-attention mechanism

After the image is divided into multiple patches, the self-attention mechanism estimates relationships among all patch representations and allows the model to assign different levels of importance to different interactions. This is achieved by transforming the image into three matrices: the Query (*Q*), Key (*K*), and Value (*V*). These matrices are computed from the input token embeddings. The query matrix *Q* represents the queries used to compare each token with other tokens, the key matrix *K* provides the corresponding comparison keys, and the value matrix *V* contains the feature information aggregated according to the resulting attention weights. The self-attention feature map can then be calculated as illustrated in Equation 8.


$$\begin{array}{c} \text{Self}\text{-Attention}\left(Q,K,V\right)=\text{softmax}\left(\frac{QK^{T}}{\sqrt{d_{q}}}\right)V, \end{array}$$ (8)

where *d*_*q*_ represents the input embedding dimension. With three attention heads, the model can represent multiple types of relationships among image patches in parallel.

The model evaluation protocol was designed based on five-fold cross-validation, such that in each iteration, four-fold were used for training and the remaining fold for evaluation. The proposed AOViT process is divided into two main stages: the first stage involves training the model within a five-fold cross-validation framework, while the second stage is dedicated to performance evaluation and an analysis of the model's explainability. These three stages are summarized in Algorithms 1–3, respectively. After the five folds are defined in Algorithm 1, Algorithm 2 is executed independently for each fold, producing five fold-specific models, each trained on four folds and evaluated on the remaining fold. This structure ensures that the evaluation of each fold is performed using a model trained solely on the remaining four-fold, thereby preventing data leakage between the training and evaluation sets.

Algorithm 2 Fold-wise training and model selection of the proposed AOViT model.

 **Require:**  Initialized AOViT model, training set, and
  evaluation fold *D*_*k*_ 
**Ensure:**  Trained AOViT checkpoint after the predefined
  20-epoch schedule for fold *k* 
1:  **for** *e* = 1 to 20 **do** 
2:   **for** each mini-batch ℬ in the training set **do** 
3:      Divide each image into non-overlapping 16 × 16
  patches using Equation 3
4:     Flatten patches and project them into the
  *E*-dimensional embedding space 
5:     Generate patch embeddings using Equation 5
6:     Add the learnable positional embeddings to
  the token sequence 
7:     Prepend the learnable class token and
  construct the ViT input sequence using Equation 7
8:     **for** *l* = 1 to *L* **do** 
9:      Compute multi-head self-attention using
  Equation 8
10:      Apply residual connection and
  normalization 
11:      Apply the feed-forward network with GELU
  activation using Equation 7
12:      Apply residual connection and
  normalization 
13:     **end for** 
14:     Extract the final class-token
  representation 
15:     Pass the representation through the
  classification head 
16:     Compute class probabilities using SoftMax 
17:     Compute cross-entropy loss 
18:     Update model parameters using AdamW
  according to Equation 4
19:   **end for** 
20:   Update the learning rate using Equation 2
21:  **end for** 
22:  Store the final checkpoint after epoch 20 for
    fold *k* 
23:  Return the trained fold-specific AOViT model

Algorithm 3 Evaluation and explainability procedure of the proposed AOViT model.

**Require:**  Five trained fold-specific AOViT models
**Ensure:**  Aggregated classification and explainability
  results
 1: **for** *k* = 1 to 5 **do**
2:   Load the final 20-epoch AOViT checkpoint for
  fold *k*
3:   Evaluate the model on evaluation fold *D*_*k*_
4:   Obtain predicted class labels for all samples
  in *D*_*k*_
5:   Compute fold-wise accuracy
6:   Compute fold-wise precision
7:   Compute fold-wise recall
8:   Compute fold-wise F1-score
9:   Store all fold-specific evaluation metrics
10:    Generate Grad-CAM explanations for selected
   samples from evaluation fold *D*_*k*_
11:    Compute Deletion AUC for the generated
   attribution maps
12:    Compute Insertion AUC for the generated
   attribution maps
13:    Store fold-specific explainability results
14: **end for**
15: Compute the mean and standard deviation of
    accuracy across the five-fold
16: Compute the mean and standard deviation of
    precision across the five-fold
17: Compute the mean and standard deviation of recall
    across the five-fold
18: Compute the mean and standard deviation of
    F1-score across the five-fold
19: Aggregate Grad-CAM and faithfulness results
    across-fold
20: Return the final five-fold cross-validation
    performance and explainability results

## 5 Results

Section 5 presents the experimental results of the proposed AOViT model on the Persian Miniature dataset. The results are organized into four subsections: overall performance and statistical stability (Section 5.1), an ablation study of the training components (Section 5.2), comparative evaluation against CNN and hybrid ViT architectures (Section 5.3), and model explainability analysis (Section 5.4).

### 5.1 Statistical analysis and result stability

Given the relatively limited size of the dataset, relying on a single train-test split could make performance estimates dependent on a specific partition. Therefore, the primary model evaluation was conducted using five-fold cross-validation. The dataset was divided into five-fold; in each iteration, four-fold were used for training and the remaining fold for evaluation. This process was repeated five times, ensuring that each sample served as part of an evaluation fold exactly once. Consequently, the paper's main results are not based on a fixed held-out test set but are derived from the model's performance across the five evaluation-fold. Metrics including accuracy, precision, recall, and F1-score were calculated independently for each fold and subsequently reported as mean and standard deviation values.

The results in Table 6 indicate relatively consistent performance across the five evaluation folds, with limited fold-to-fold variation. To more precisely characterize the uncertainty in model performance, a 95% confidence interval was calculated for the primary metrics in addition to the mean and standard deviation. Based on the five-fold results, AOViT achieved a mean accuracy of 0.9385 ± 0.0079, precision of 0.9394 ± 0.0084, recall of 0.9385 ± 0.0079, and F1-score of 0.9384 ± 0.0083. Using the *t*-distribution and the five fold-level values, the approximate 95% confidence intervals were calculated as 0.9287–0.9483 for accuracy, 0.9290–0.9498 for precision, 0.9287–0.9483 for recall, and 0.9281–0.9487 for the F1-score.

**Table 6:** Cross-validation performance and uncertainty of the proposed AOViT model.

Metric	Mean	SD	Approx. 95% CI
Accuracy	0.9385	0.0079	0.9287–0.9483
Precision	0.9394	0.0084	0.9290–0.9498
Recall	0.9385	0.0079	0.9287–0.9483
F1-score	0.9384	0.0083	0.9281–0.9487

These intervals should be interpreted as an indicator of performance variability across folds. Since the training sets in cross-validation overlap, the fold results are not entirely independent; consequently, these confidence intervals should not be interpreted as a fully independent estimate of population uncertainty. Nevertheless, they provide useful supplementary information regarding the stability of the model's performance across different partitions.

The comparisons with baseline and hybrid models reported in the subsequent sections are descriptive unless otherwise stated. A paired inferential comparison, such as McNemar's test, requires sample-level predictions from both classifiers for the same evaluation instances. Because such paired prediction records are not reported in the present analysis, no McNemar or paired-bootstrap significance results are claimed. Accordingly, numerical differences between models are interpreted as descriptive performance differences rather than evidence of statistical significance.

### 5.2 Ablation study

To examine the contribution of individual training components, an ablation study was conducted under the same five-fold cross-validation setting. Across ablation configurations, the common experimental settings were kept fixed while the selected training component was varied.

The ablation results are summarized in Table 7, which compares the baseline ViT model, the ViT model with data augmentation, the ViT model with a learning rate scheduler, the ViT model with the AdamW optimizer, and the full proposed model with all components, i.e., AOViT. Ablation results indicated that data augmentation alone did not lead to improved performance. Specifically, accuracy dropped from 91.73% in the baseline ViT to 90.73% in the configuration that included augmentation. Therefore, augmentation in this study should not be interpreted as an independent factor for accuracy improvement. Its inclusion in the final configuration was primarily intended for regularization and increasing training data diversity, alongside other optimization components. Since the current ablation study did not separately evaluate all pairwise and higher-order interactions between augmentation, AdamW, and the scheduler, the independent or synergistic contribution of augmentation to the full model cannot be definitively determined. Based on the observed numerical results, the largest individual improvement was associated with the use of AdamW.

**Table 7:** Ablation study results.

Configuration	Accuracy (%)
Baseline ViT	91.73
Baseline ViT + data augmentation	90.73
Baseline ViT + learning rate scheduler	91.62
Baseline ViT + AdamW	93.18
Full proposed model	93.85

The learning-rate-scheduler configuration achieved an accuracy of 91.62% and an F1-score of 91.58%, which were slightly lower than the baseline values. In contrast, the AdamW configuration achieved 93.18% accuracy and an F1-score of 93.12%, representing the largest numerical improvement among the individually evaluated components.

When all selected components were combined in the final AOViT configuration, the model achieved 93.85% accuracy and an F1-score of 93.81%, representing the highest performance among the ablation configurations reported in this study.

### 5.3 Comparative evaluation for the proposed AOViT model

Section 5.3 compares the proposed AOViT model with several CNN architectures, including InceptionV3, VGG-19, VGG-16, EfficientNet-B4, and Xception. These comparative experiments were conducted under the preprocessing, augmentation, and training settings described in Tables 3, 4.

As shown in Figure 3, AOViT achieved strong comparative performance across the reported classification metrics. Its self-attention mechanism enables interactions among spatially distant patches, providing a direct mechanism for modeling global contextual relationships alongside local visual cues such as texture and color. CNNs, by contrast, build representations through hierarchically expanding receptive fields.

**Figure 3:**
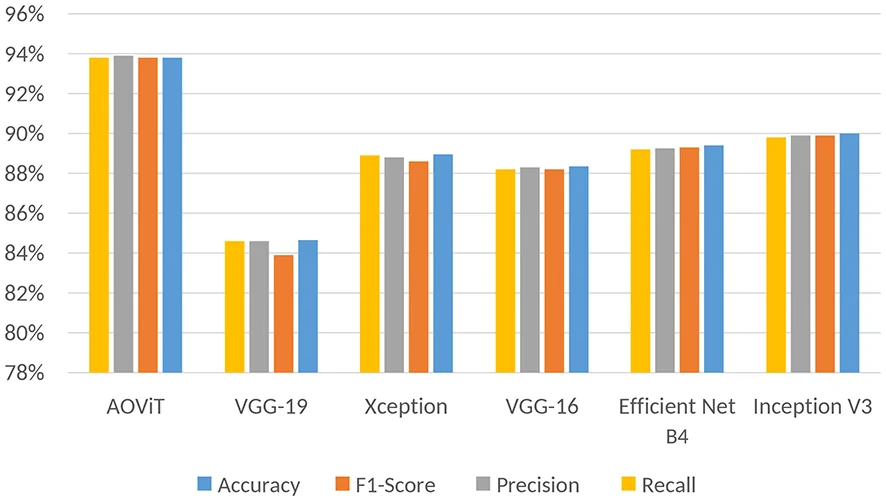
Comparative performance of the proposed AOViT model and CNN variants.

To further evaluate the proposed AOViT model, three hybrid architectures were designed using ViT with InceptionV3, ResNet50, and EfficientNet backbones, as illustrated in Table 8. The proposed model achieved an accuracy 2.85 percentage points higher than the strongest hybrid model in this comparison, ViT + InceptionV3.

**Table 8:** Descriptive performance comparison of AOViT and comparative hybrid models.

Model	Accuracy (%)	Precision	Recall	F1-score	Δ Accuracy vs. AOViT (pp)
The proposed AOViT	93.85	0.9394	0.9385	0.9384	–
ViT + InceptionV3	91.00	0.90	0.91	0.91	–2.85
ViT + ResNet50	86.67	0.87	0.87	0.87	–7.18
ViT + EfficientNet-B4	82.22	0.84	0.83	0.83	–11.63

A descriptive comparison indicates that AOViT achieved higher accuracy than the hybrid models examined. The differences in absolute accuracy compared to ViT + InceptionV3, ViT + ResNet50, and ViT + EfficientNet-B4 were 2.85, 7.18, and 11.63 percentage points, respectively. However, these numerical differences alone should not be interpreted as evidence of statistical significance. To test for statistical significance between two classifiers evaluated on the same samples, sample-level predictions are required to identify the number of discordant samples and perform a paired McNemar's test.

The backbones, architectural strategies, and trainable parameter counts of the hybrid models are summarized in Table 9.

**Table 9:** Architectural characteristics and trainable parameters of AOViT and comparative hybrid models.

Model	Backbone	Architectural/training strategy	Trainable parameters
ViT + InceptionV3	InceptionV3	Hybrid Transformer–CNN architecture with multi-scale convolutional feature extraction	109
ViT + ResNet50	ResNet50	Hybrid Transformer–CNN architecture with residual convolutional feature extraction	112
ViT + EfficientNet	EfficientNet-B4	Hybrid Transformer–CNN architecture with parameter-efficient convolutional feature extraction	105
AOViT	ViT-Tiny/16	Lightweight Vision Transformer with end-to-end fine-tuning, AdamW optimization, and cosine learning-rate scheduling	5.53

The relatively low number of learnable parameters in AOViT stems from the use of the lightweight ViT-Tiny/16 configuration and should not be directly compared to the larger ViT-Base architecture. In the configuration employed, 224 × 224 images are divided into 16 × 16 patches, the embedding dimension is 192, and the architecture comprises 12 Transformer encoder blocks and three attention heads. With a five-class classification head, the model has approximately 5.53 million learnable parameters. Thus, the parameter count reported in Table 9 reflects the use of a lightweight ViT-Tiny architecture rather than the truncation or unspecified removal of ViT-Base layers. Furthermore, AOViT required less training time than all evaluated hybrid models, although the baseline ViT trained faster. Table 10 shows the training time for the proposed AOViT and other hybrid models.

**Table 10:** Training time for the proposed AOViT and other hybrid models.

Model	Training time (s)	Reason
Baseline ViT	528.311	Only the transformer is trained. No additional CNN backbone is included.
ViT + ResNet50	812.742	ResNet50 adds convolutional layers before the transformer, increasing FLOPs and the number of parameters.
ViT + Xception	883.453	Xception uses depthwise separable convolutions and a deeper feature extraction process than ResNet50, increasing computational requirements.
ViT + Inception v3	957.721	Multiple parallel convolution branches make the Inception architecture computationally expensive.
ViT + EfficientNet	1034.309	EfficientNet simultaneously scales depth, width, and resolution, resulting in higher computational cost.
AOViT	659.294	Data augmentation introduces preprocessing overhead, while AdamW and the learning rate scheduler add minor optimization costs.

It should be noted that the comparison with results from previously published studies is purely descriptive in nature. Given that these studies employed different datasets, preprocessing procedures, data partitions, and experimental protocols, and since shared sample-level predictions for the same samples are unavailable, conducting a paired statistical test or calculating a direct *p*-value between AOViT and prior studies would not be methodologically valid. Therefore, the numerical differences reported in comparison to prior work are interpreted solely as performance comparisons and should not be regarded as evidence of statistically significant superiority.

### 5.4 AOViT model analysis

Section 5.4 uses Grad-CAM as an XAI technique to examine the image regions associated with the predictions of the proposed AOViT model. The resulting attribution maps are interpreted qualitatively and are complemented by quantitative faithfulness measures based on Deletion and Insertion AUC.

The Grad-CAM visualization for AOViT is shown in Figure 4. Warmer colors (red and yellow) indicate regions with higher attribution intensity, whereas green, cyan, and blue indicate progressively lower attribution values. Three thresholds (τ = 0.3, 0.5, 0.7) were applied to the Grad-CAM map to illustrate how the selected active regions change with the threshold, as shown in Figure 5. Areas enclosed by the green contours correspond to activations exceeding the selected threshold.

**Figure 4:**
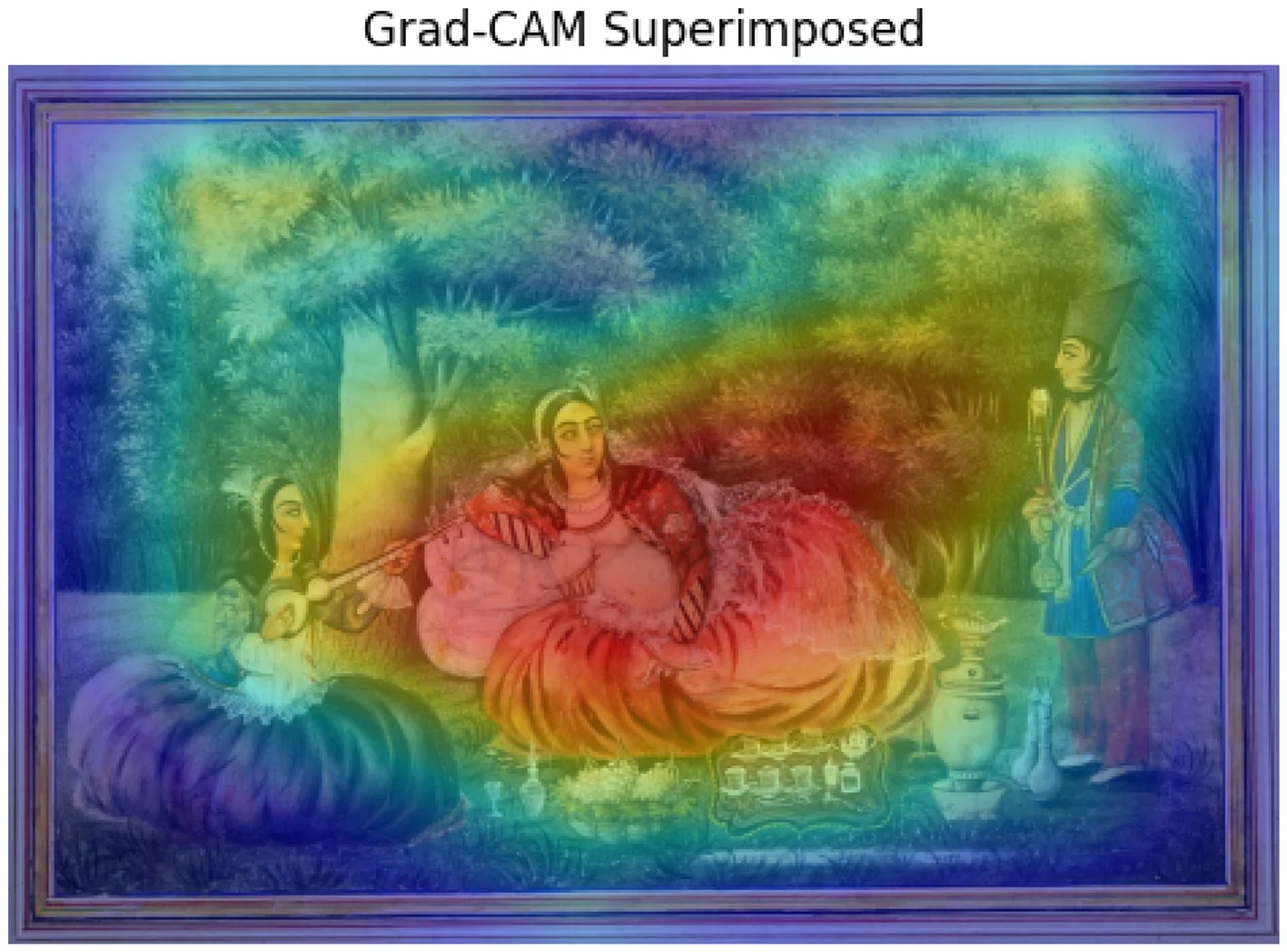
The proposed AOViT model Grad-CAM.

**Figure 5:**
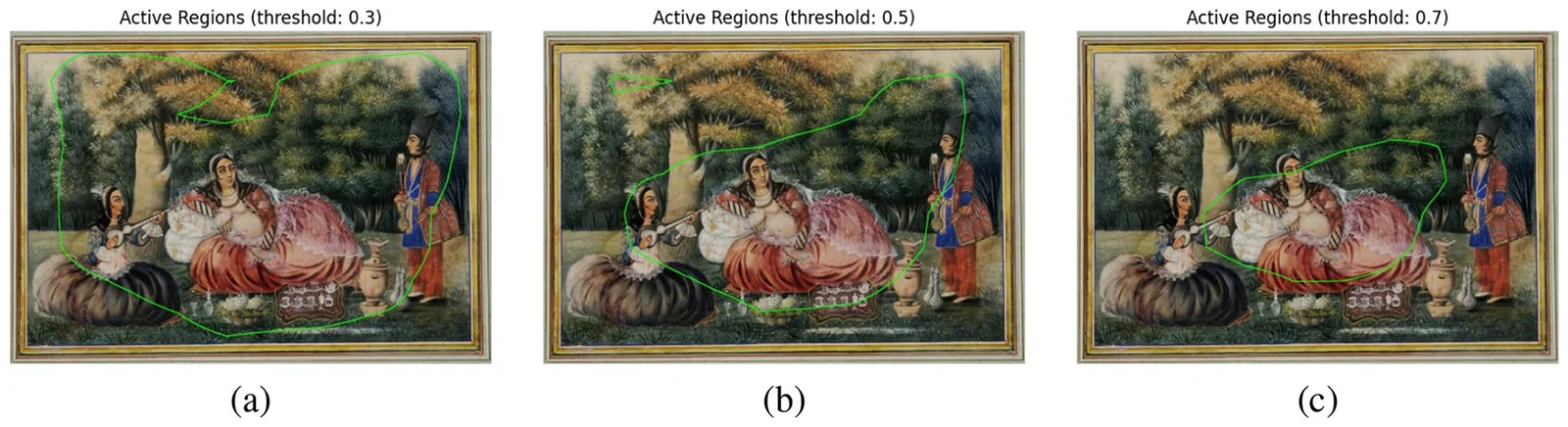
Grad-CAM threshold visualizations for the proposed AOViT model at different activation thresholds: **(a)** τ = 0.3, **(b)** τ = 0.5, and **(c)** τ = 0.7.

The Grad-CAM visualization provides a spatial representation of image regions associated with the model prediction. The greatest activation is localized in the lower left foreground and in the middle of the painting as depicted in Figure 4. The most striking response can be seen as a large warm red patch that covers the small seated figure and the vessel nearby, and a larger patch of warm red, orange and yellow activations that stretches over the central figure. The surrounding foliage is moderately activated with green and cyan colors, while the figure standing to the right, the decorative frame and the image corners have relatively low activations and show more blue colors.

The observed activation pattern suggests that, for this example, the model's prediction is influenced primarily by the central human figures and distinctive foreground objects, whereas the background and decorative frame receive lower attribution. Figure 5 shows the effect of threshold selection. For this value of τ = 0.7, only the highest activated regions are kept in Figure 5c. The contour excludes the standing figure, most of the background vegetation, and the decorative frame, while retaining the central seated figure, the lower-left foreground hotspot, and nearby objects. These regions therefore represent the highest-attribution areas under this threshold for the illustrated example.

At τ = 0.5 (Figure 5b), the contour expands to include regions with moderate attribution, including part of the vegetation in the upper-left area and regions extending toward the standing figure on the right. At the lower threshold of τ = 0.3 Figure 5a), additional low-attribution regions are incorporated, causing the contour to cover a much larger portion of the painting, including parts of the background and frame. Thus, Figure 5a illustrates the broad coverage produced by a low threshold, whereas Figure 5c isolates only the highest-attribution regions. Figures 6–8 show Grad-CAM visualizations for the baseline ViT, VGG-16, and VGG-19 models, respectively. The attribution patterns differ across architectures, reflecting differences in their learned representations.

**Figure 6:**
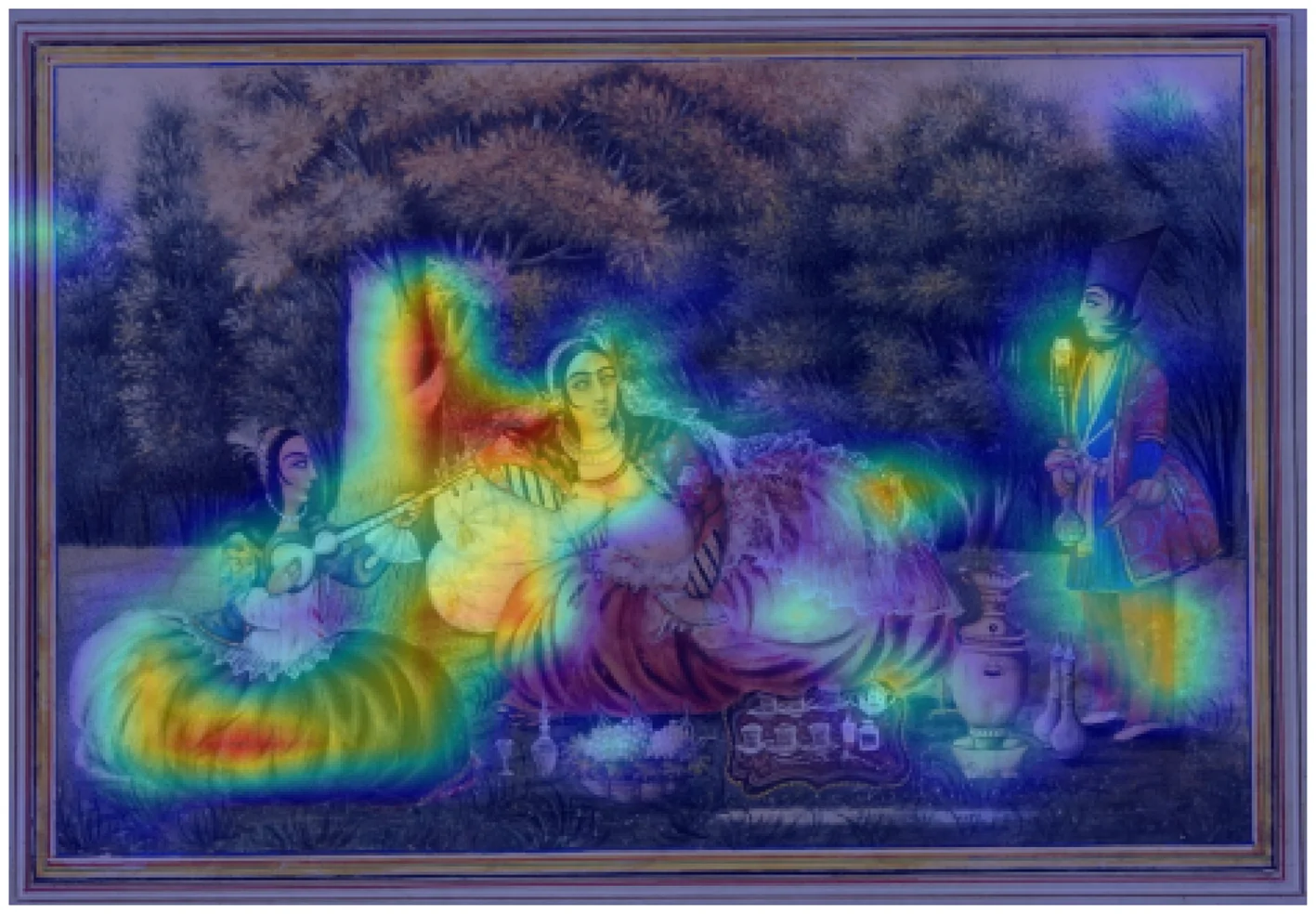
Grad-CAM visualization superimposed on the input image for the baseline ViT.

Figure 6 shows a comparatively diffuse Grad-CAM pattern for the baseline ViT, with warm activations distributed across the left musician, central figure, standing figure on the right, and several background regions. This visualization suggests that the baseline model distributes attribution across multiple spatial regions rather than concentrating it within a single localized area.

The VGG-16 and VGG-19 visualizations in Figures 7, 8 show sharper but more spatially fragmented attribution patterns. VGG-16 distributes higher-attribution regions across the lower foreground and several objects, whereas VGG-19 places greater emphasis on the central group while still producing multiple disconnected regions. These qualitative differences are interpreted cautiously because visual concentration alone does not establish explanation faithfulness or localization accuracy.

**Figure 7:**
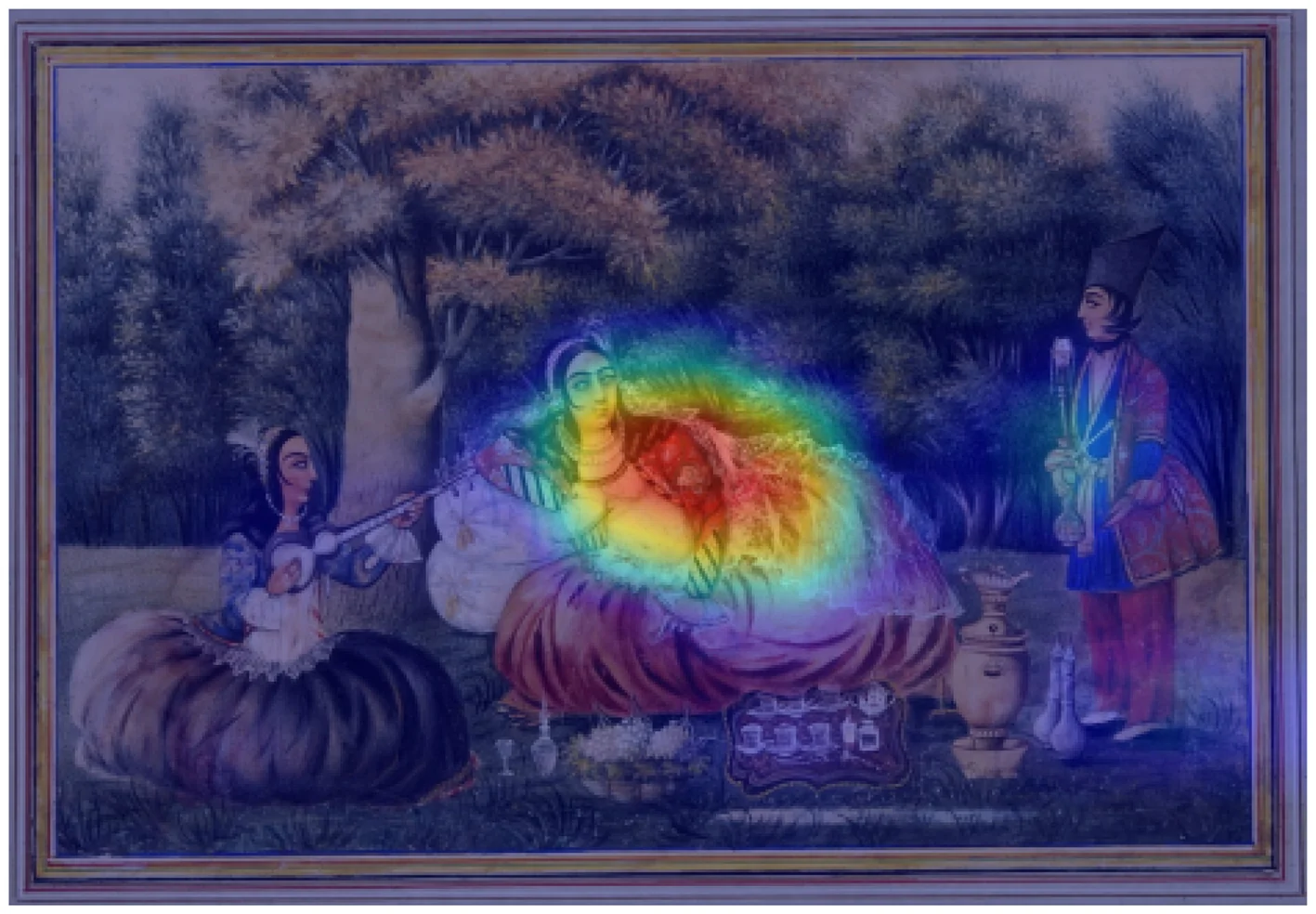
Grad-CAM visualization superimposed on the input image for VGG-16.

**Figure 8:**
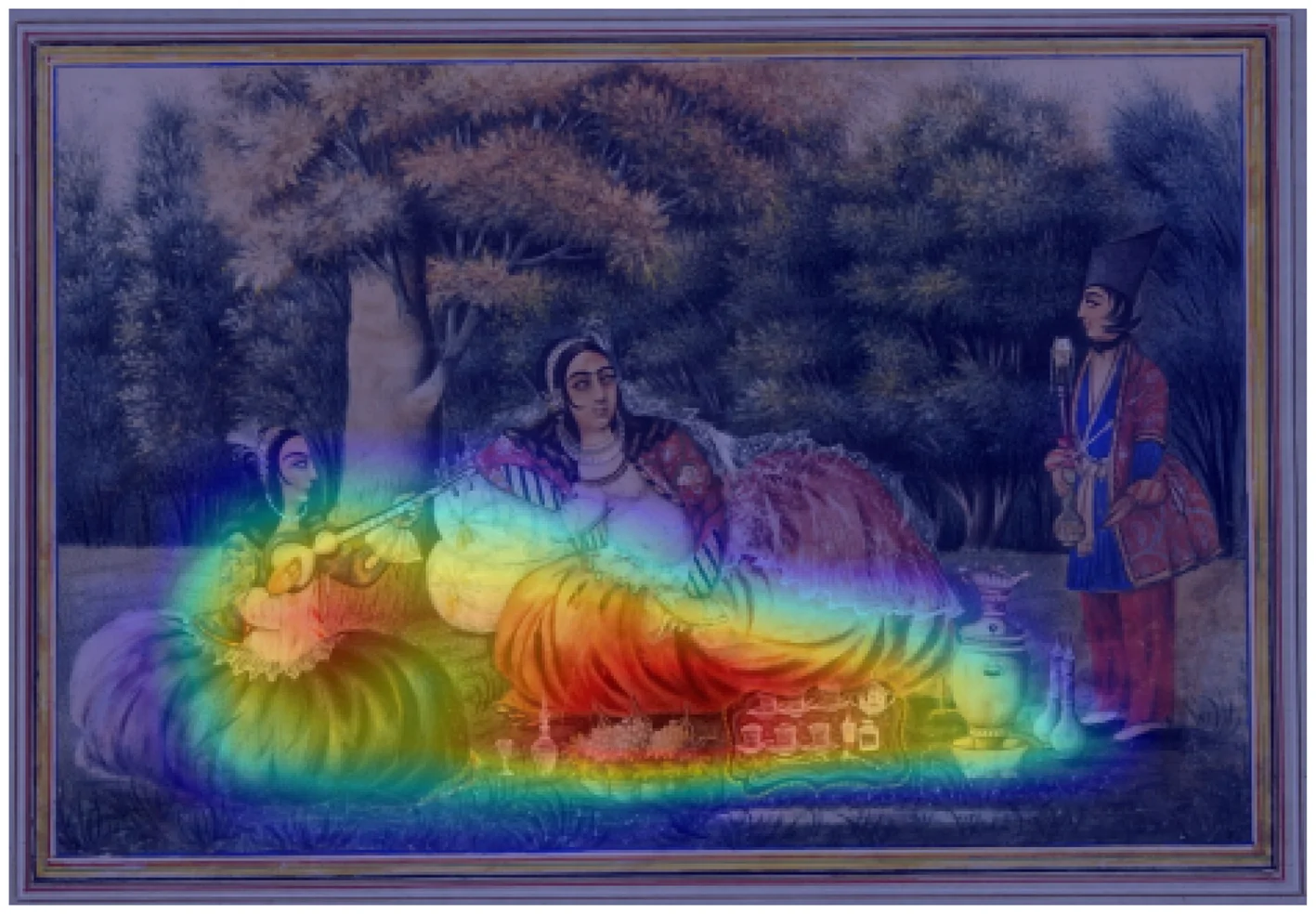
Grad-CAM visualization superimposed on the input image for VGG-19.

Grad-CAM visualizations were employed to provide a qualitative interpretation of the regions influencing the model's decision. However, the evaluation of explainability was not based solely on visual inspection. To quantitatively measure the faithfulness of the attribution maps, Deletion AUC and Insertion AUC were used (Belhraka and Belhrak, 2026). Deletion AUC assesses the rate at which the model's confidence drops as regions with the highest attribution scores are progressively removed; thus, a lower value is preferable. Conversely, Insertion AUC measures the rate at which model confidence is restored as important regions are progressively added to a baseline image; consequently, a higher value indicates greater faithfulness. However, these two metrics should not be confused with spatial accuracy relative to ground-truth regions. Since the dataset used in this study lacks reference spatial annotations for stylistic elements, metrics such as IoU or pointing-game accuracy could not be calculated. Therefore, in this study, deletion and insertion AUC measure the faithfulness of the explanations rather than absolute localization accuracy. Table 11 summarizes the Deletion and Insertion AUC values for the evaluated models. As shown in Table 11, AOViT achieved the lowest Deletion AUC (0.118) and the highest Insertion AUC (0.642) among the evaluated models. Its corresponding standard deviations were ±0.029 and ±0.039, respectively. Relative to the baseline ViT (Deletion AUC 0.182; Insertion AUC 0.471), these values indicate stronger attribution faithfulness under the adopted deletion/insertion evaluation protocol. VGG-16 and VGG-19 also achieved more favorable faithfulness scores than the baseline ViT. These results quantify explanation faithfulness, but they should not be interpreted as direct measurements of spatial localization accuracy because ground-truth region annotations are unavailable.

**Table 11:** Deletion and insertion AUC for the grad-CAM attribution maps.

Model	Deletion AUC	Insertion AUC
Baseline ViT	0.182 ± 0.041	0.471 ± 0.053
VGG-16	0.156 ± 0.037	0.538 ± 0.048
VGG-19	0.149 ± 0.035	0.561 ± 0.046
The proposed AOViT	0.118 ± 0.029	0.642 ± 0.039

## 6 Conclusion

Artistic-style classification is an important task in digital cultural heritage because it can support the assignment of artworks to historical and stylistic contexts and contribute to preservation, restoration, education, and collection analysis. In this study, AOViT was developed for artistic-style classification of Persian heritage paintings. The proposed framework combines transfer learning, data augmentation, AdamW optimization, and learning-rate scheduling within a lightweight ViT-Tiny/16 architecture. Under five-fold cross-validation, AOViT achieved a mean accuracy of 93.85% on the Persian Miniatures dataset, with relatively limited variation across evaluation folds. The model also achieved competitive performance relative to the evaluated CNN-based architectures, including VGG-16, VGG-19, InceptionV3, EfficientNet-B4, and Xception. The ablation study further showed that AdamW provided the largest individual numerical improvement among the evaluated training components, whereas data augmentation and the learning-rate scheduler did not independently improve accuracy relative to the baseline configuration. Several limitations should be considered when interpreting these findings. The dataset is relatively small and domain-specific, which limits the extent to which the results can be generalized to larger and more diverse artistic collections. Although five-fold cross-validation was used to reduce dependence on a single data partition, cross-dataset validation was not performed. In addition, the computational requirements of Transformer-based models may constrain deployment in real-time or resource-limited heritage applications. Future work should therefore evaluate the framework on larger and more diverse datasets, investigate cross-dataset generalization, and explore lightweight or hierarchical Transformer architectures and efficient CNN–Transformer hybrids. Additional quantitative explainability analyses and computational optimization strategies should also be considered to strengthen the interpretability and practical applicability of the framework.

## Data Availability

The original contributions presented in the study are included in the article/supplementary material, further inquiries can be directed to the corresponding authors.
